# Integration of pharmacist independent prescribers into general practice: a mixed-methods study of pharmacists’ and patients’ views

**DOI:** 10.1186/s40545-023-00520-9

**Published:** 2023-01-19

**Authors:** Abdullah A. Alshehri, Ali M. K. Hindi, Ejaz Cheema, M. Sayeed Haque, Zahraa Jalal, Asma Yahyouche

**Affiliations:** 1 Clinical Pharmacy Department, College of Pharmacy, Taif University, Al Huwaya, Taif, 26571 Saudi Arabia; 2 School of Pharmacy, College of Medical and Dental Sciences, University of Birmingham, Edgbaston, Birmingham, B15 2TT UK; 3 Division of Pharmacy and Optometry, School of Health Sciences, Faculty of Biology, Medicine and Health, The University of Manchester, Manchester, UK; 4 School of Pharmacy, University of Management and Technology, Lahore, Pakistan; 5 Institute of Applied Health Research, College of Medical and Dental Sciences, University of Birmingham, Edgbaston, Birmingham, B15 2TT UK

**Keywords:** Clinical pharmacist, Practice-based pharmacist, Pharmacist independent prescriber, General practice, Primary health care, Family practice

## Abstract

**Background:**

Since 2015, the National Health Service (NHS) has funded pharmacists to work in general practice (GP practice) to ease workload pressures. This requires pharmacists to work in new roles and be integrated effectively in GPs. Independent prescribing is a key part of the GP pharmacist role, but little is known about pharmacists’ integration into GP practice as well as patients’ perceptions and experiences of the care provided by GP pharmacists. This study aims to explore the perceptions of pharmacist independent prescribers (PIPs) about their integration into GP practice and gain insight into patients’ perceptions about the care provided to them by pharmacists.

**Methods:**

A mixed-methods study comprising semi-structured interviews with PIPs (*n* = 13) followed by questionnaire-based assessment of patients’ (*n* = 77) evaluation of pharmacists’ care was conducted between December 2019 and March 2020. Quantitative data were analysed using descriptive statistics. Interviews and open comments of the survey were thematically analysed.

**Results:**

Pharmacist independent prescribers reported undertaking a range of patient-facing and non-clinical roles. Lack of understanding about PIPs’ clinical role and working beyond their clinical area of competence were some of the barriers to their integration into GP practice. Most patients were satisfied with the consultations they received from pharmacists and reported confidence in the pharmacist’s recommendations about their health conditions. However, a few patients (14%) felt they would still need to consult a general practitioner after their appointment and 11% were not sure if a further consultation was needed.

**Conclusions:**

Pharmacist independent prescribers provide a range of clinical services for the management of long-term conditions which appear to be recognised by patients. However, there is a need to address the barriers to PIPs’ integration into GP practice to optimise their skill-mix and patient-centred care.

**Supplementary Information:**

The online version contains supplementary material available at 10.1186/s40545-023-00520-9.

## Background

The increased number of patients with multimorbidity coupled with the associated increase in medication use has led to a major increase in GP practice workload worldwide [1–3]. In the United Kingdom (UK), the National Health Service (NHS) has been experiencing a workforce crisis due to difficulties in recruitment, retention, and early retirement of GP practitioners [4, 5]. Evidence from self-reported surveys suggest an association between increased GP practice workload and decreased patient satisfaction [6–8].

Consequently, the NHS has taken several policy initiatives to expand the role of non-medical healthcare professionals, such as nurses and pharmacists [9, 10]. The "clinical pharmacists in general practice" scheme was launched in 2015 by NHS England (NHSE) in different phases with the aim of recruiting and training thousands of pharmacists to be independent prescribers primarily responsible for providing clinical services to patients [10–12]. Since the introduction of the scheme, the number of registered pharmacist independent prescribers (PIPs) in England has steadily increased by more than threefold from 2224 in 2016 to 7348 in 2020 [13]. Moreover, the General Pharmaceutical Council (GPhC) has recently implemented new standards to ensure pharmacist students develop the skills and knowledge required to become pharmacist independent prescribers at the time of registration with the GPhC [14]. To date, most of the practice-based pharmacists are independent prescribers, and their services are focused on medication reviews, management of long-term medical conditions and minor ailments, telephone consultations for follow-up and prescribing [15–19]. Evidence from previous studies that involved patients with long-term medical conditions reported that GP pharmacist-led consultations could provide patients with better access to GP practice services [20, 21]. Other evidence suggests that GP pharmacists help reduce general practitioners’ workloads and enable them to focus on patients suffering from complex conditions [22, 23].

Unlike in hospital and community pharmacy settings, PIPs in GP practice is a relatively new role which is not well-established. For instance, some PIPs may be provided with opportunities to take on patient-facing clinical roles, whereas others may be mainly confined to administrative work, depending on the needs of the GP practice [15, 20, 24, 25]. Moreover, similar to other non-medical healthcare professionals, pharmacists may experience uncertainty as to their professional role and identity in the GP practice [26, 27]. Recent policy initiatives such as the NHS long-term plan and the Pharmacy Integration Fund aim to increase the number of PIPs in GP practice nationwide. It is, therefore, important to understand how to facilitate the integration of PIPs in GP practice to ensure optimal skill-mix and service provision.

To date, a few studies have evaluated the integration of pharmacists in GP practice [15, 17]. One study investigated the role and integration of GP pharmacists at the early stage of the NHSE scheme, where the number of PIPs was relatively small and the clinical pharmacist role was less developed [15]. Another study which evaluated the satisfaction of pharmacists with their integration into GP practice suggested a lack of knowledge about the current practice in England relative to the potential barriers of PIPs’ integration into GP practice [17]. However, little is known about patients’ experiences and satisfaction with the services provided by PIPs in GP practice [28]. Previous studies looking at patient experiences and satisfaction with the services provided by pharmacists in primary care have been limited to non-patient facing roles [29, 30]. Patients’ perceptions and experiences with GP practice services are an important outcome of medical care and a determinant of care quality [31–33]. Patients who are more satisfied with the services provided are more likely to comply with treatment and better adherence and compliance leading to greater health outcomes and continuity of care [34, 35]. This study, therefore, aims to explore the integration of PIPs into GP practice and patients’ satisfaction with consultations provided by PIPs within GP practices in England.

## Methods

### Study design

A mixed-methods study comprising semi-structured interviews with PIPs and a questionnaire-based service evaluation of patients who had consultations with the interviewed PIPs was conducted between December 2019 and March 2020.Using a mixed-methods approach enabled the coverage of a broader sample frame within a limited timeframe [36, 37]. In our study, qualitative interviews provided an in-depth exploration of factors influencing the integration of PIPs into GP practice, while patient surveys helped supplement the qualitative findings by providing insights from patients who received care from PIPs. Hence, we were able to provide more multi-faceted conclusions relative to the integration of PIPs into GP practice.

### Semi-structured interviews

An interview schedule was developed following a review of the previous literature and discussion among the research team. The interview schedule was pilot tested with two GP pharmacists. The interviews were expected to take 30–50 min.

Convenience sampling was used for recruitment. Advertisements and study invitation letters were disseminated through practice-based pharmacists’ training days, professional events and social media groups. Participants willing to participate were asked to email the lead researcher, AA. All interviews were conducted by AA either face-to-face or by phone until data saturation was reached. All interviews were audio recorded and transcribed verbatim by an approved professional transcription service.

### Questionnaire-based service evaluation

The questionnaire was developed following previous literature and discussion between the research team (see Additional file 1: S1). Following consultations with a PIP, the questionnaire was piloted on five patients.

All PIPs who participated in the interviews were invited to distribute the questionnaires and participant information sheets to their patients. Patients who were willing to participate completed the questionnaire and handed it to reception. The lead researcher, AA, held regular bi-weekly meetings with PIPs to discuss progress and collect completed questionnaires.

### Data analysis

Qualitative data from semi-structured interviews and open-ended questions in the survey were inductively analysed using thematic analysis. These processes were performed by AA and then revised by AY independently. The final themes and codes were agreed upon by the research team. NVivo 12, QSR International aided the management of data analysis. Qualitative data from the questionnaires were organised manually within a Microsoft Excel spreadsheet. Quantitative data from the questionnaire were entered into SPSS (version 26) and double-checked by another member of the research team. The data were summarised descriptively.

## Results

### Participant characteristics

#### Pharmacist independent prescribers

Thirteen PIPs agreed to participate in the interviews. Nine interviews were conducted face-to-face and four by telephone. Pharmacist independent prescribers’ characteristics and their working status are presented in Table 1.

**Table 1:** Pharmacists’ characteristics and their working status

Participant ID	Gender	General practice site	Specialty	Years in GP practice	Working Status	Number of participants who filled the questionnaire after attending PIPs’ appointment
01	Female	Dudley	PIP in Diabetes	20 years	Part time	7
02	Male	Wolverhampton	PIP in Hypertension	6 years	Part time	Did not participate
03	Female	Leicester	PIP in Hypertension	3 years	Full time	Did not participate
04	Male	Walsall	PIP in Diabetes	2 years	Full time	11
05	Male	Birmingham	PIP in Hypertension	25 years	Full time	5
06	Female	South Lakeland	PIP in Diabetes	3 years	Full time	23
07	Female	Portsmouth	PIP in Hypertension	2 years	Full time	Did not participate
08	Male	Birmingham	PIP in Diabetes	8 months	Part time	20
09	Female	Brighton and Hove	PIP in sexual health	4 years	Full time	Did not participate
10	Male	Kent	PIP in Diabetes	4 years	Full time	Did not participate
11	Female	Dudley	PIP in Hypertension	3.5 years	Full time	Did not participate
12	Female	Hampshire	PIP in Hypertension	4 years	Full time	10
13	Female	Milton Keynes	PIP in Diabetes	2.5 years	Full time	9
Total of received questionnaire						85

#### Patient questionnaire

A total of 77 patients who received consultations from seven different GP practices completed the questionnaire (Table 2). The reported length of appointments ranged from 5 to 40 min, with an average (mean) duration of 17 min (SD = 7.5). Less than half of patients (47%, *n* = 36) had received only one form of advice or intervention, of whom the vast majority (*n* = 33) said that this was related to medication only.

**Table 2:** Patients’ characteristics

Characteristic	*N* (*N* = 77)	%
Gender		
Male	33	42.9
Female	43	55.8
Prefer not to say	1	1.3
Age		
Minimum = 40 Maximum = 85 Mean = 63.42 SD = 11.2 Median 63 (interquartile range = 16)		
Type of health conditions that patients had attended for		
Hypertension	36	46.7
Diabetes	15	19.4
Other or mixed condition	26	33.7
Length of appointments		
Less than 10 min	3	3.9
10–19 min	44	57.1
20–29 min	21	27.2
30–39 min	6	7.8
More than 40 min	3	3.9
Number of previous appointments with GP pharmacists (note: there were 75 participants, not 77, who completed this question)		
No appointment before	16	21.3
1	14	18.6
2	18	24
3–5	16	21.3
6–10	8	10.6
More than 10	3	4
Types of services provided by PIPs		
Medication only	33	42.8
Medication and lifestyle	16	20.7
Medication, lifestyle and disease	15	19.5
Medication and disease	3	3.9
Other	10	13

Eight patients excluded due to missing data

### PIPs’ perception of their role and integration

The final themes and sub-themes that resulted from thematic analysis are shown in Table 3.

**Table 3:** Themes identified from PIPs during interviews

Themes	Sub-themes
Emerging roles of PIPs in general practice	•Patient-facing roles •Non-clinical roles
Barriers and facilitators to integration into general practice	•Understanding and recognition of PIP role •Formal and informal training and development •Interpersonal skills of PIP

#### Theme 1: emerging roles of PIPs in general practice

This theme covers information provided by the participants on the roles they performed in GP practice.

##### Patient-facing roles

Patient-facing roles involved medication reviews and general health checks as part of patient clinics. Most interactions were carried out in person, with telephone calls mostly used for following up. Medication reviews were often a central focus of patient consultations conducted by participants that reflected their unique expertise in this area. Many reported wide-ranging responsibilities which included diagnosis and management of acute and chronic conditions, risk assessments, physical assessments and referrals to other healthcare professionals.

“I do patient clinics which includes medication reviews, poly pharmacy reviews, blood pressure checks, diabetes reviews” (PIP 10)

##### Non-patient-facing roles or administrative roles

Participants reported undertaking non-patient-facing roles and/or administrative roles in GP practice, with most of these related to medications including review of prescriptions and dealing with prescription queries. A few participants also described wider non-clinical leadership and management responsibilities which included training other staff on medication-related issues and involvement in quality improvement initiatives relative to the dispensing procedures or patient monitoring systems.

“I also do the non-urgent stuff like prescription review, prescriptions, audits, doing the best optimisation scheme, dealing with the drugs that is the most cost effective plus community effective as well and also give training to the nurses and doctors as well” (PIP 10).

However, it was evident from the interviews that the scope of patient consultations conducted by the PIPs varied depending on the needs of the GP practice as well as to the extent to which these responsibilities were shared between other healthcare professionals, such as general practitioners, nurses or healthcare workers.

#### Theme 2: barriers and facilitators to the integration of PIPs into general practice

Three main factors which enabled/hindered PIPs’ integration into GP practice are described below:

##### Understanding and recognition of PIP role

Some participants described situations, where PIPs had been asked to make decisions or carry out roles beyond their clinical competence due to GP practice teams’ lack of an understanding of their role. This was perceived to potentially jeopardise patient safety.

*“A lot of patients … will have polypharmacy, multi-morbidity and this is where the individual Pharmacist has to make their own decision as to what they’re happy to prescribe or not prescribe … I think that can cause problems because if the GPs see the Pharmacist as an Independent Prescriber and … say the Hospital asks the GP surgery to prescribe a new medication. Usually, the GPs would expect that Pharmacist to prescribe that medication but then individually the Pharmacist may feel that they’re not competent in that area.” (PIP 5)*

Role clarity was also important to ensure that PIPs and their employing practice(s) are adequately covered by indemnity insurance, thereby providing confidence to the practice that they can safely and legally perform their responsibilities.

*“Another thing you have to think about all the time is like are you practicing with your scope? Is it within your competence? Is it safe what you are doing? You know, will your indemnity cover you for what you are doing?” (PIP 11)*

Other participants reported that general practitioners remained reluctant to give up aspects of their own patient care services due to concerns about continuity in patient care.

*“The GPs are hesitant to let go of the person because they like to give them more of a holistic approach and deal with all their conditions together. So, I think a lot of pharmacists are meeting resistance in that way.” (PIP 9)*

Over time, when practice staff became more aware of /familiar with PIPs’ competence, they gave them opportunities to utilise their skills and knowledge in a manner that eased staff workload.

“It is just building the time, taking the workload off the GPs and saying, ‘I can do some of this.’ And once they realise what you can do, they give you more of it.” (PIP 11)

Some noted that patients were initially reluctant to make an appointment with the PIP, because they were not aware of the services they can provide.

*“When you mention you are a pharmacist, they think you are just something inside the GP surgery like a community pharmacy so getting their head around what a pharmacist in general practice does is a little bit of a challenge … You get some patients who really don’t know why they are seeing the pharmacist not than the GP and they would prefer to see the GP. So, it is still whether they have 100% faith in the pharmacist and it is not always the case. (PIP 11)*

After receiving a consultation, patients saw the value of having PIP consultations, such as receiving advice from an experienced healthcare professional and with longer consultation times.

*“When I start speaking to them and when they find out the knowledge and skills that I have and information I can pass on to them, they are impressed. They are happy, they’ve learnt something and they find that you’re almost somewhere between a GP and a Nurse because you have medical knowledge and you have a longer consultation. (PIP 3).*

Participants indicated that acceptance of PIPs by patients contributed to their successful integration into the practice as this provided PIPs with more opportunities to provide patient-centred services.

“You do get the odd patient who just want to see a doctor and doesn't want to see a pharmacist but generally I think patients are quite happy to see a pharmacist.” (PIP 13)

Some stressed the importance of a multidisciplinary team approach to facilitate integration of PIPs drawing on ways in which patient care was split/coordinated between different specialist staff within the practice. A multi-disciplinary approach was perceived to enhance patient care.

“There’s a number of different professions working together for one aim - to improve patient outcomes … it amalgamates all the different professional approaches into one so that we can actually develop and work towards better outcomes.” (PIP 8)

In this context, the use of organisational processes such as practice meetings or clinical meetings were perceived as being helpful to facilitate integration of PIPs. These multidisciplinary meetings provided PIPs with opportunities to demonstrate their knowledge and expertise by contributing to team discussions.

“We get to know more about what's happening in a practice and also, we can put our ideas forward as well. And feel more part of a team really.” (PIP13)

##### Formal and informal training and development

Participants who had received (ongoing/priori) formal education and training relevant to their PIP role mentioned how this had helped them develop the knowledge and skills necessary to adapt to their new roles and make an effective contribution to the practice, thus facilitating their integration.

*“There was a structured program in place … So for me it was a gradual introduction which was quite good for me because it wasn’t just a case of just being thrown into clinics, it was a case of learning the system from afar … Sort of reviewing it from a distance before getting stuck in … with the help of a CPPE [Centre for Pharmacy Postgraduate Education] Clinical Doctors Pathway” (PIP 2)*

In contrast, some participants reported having no structured training opportunities available to them and emphasised on the need for ongoing training:

*“The problem is there is no structured training at the moment and I think there needs to be. There is definitely a need for it …. So the new pharmacists if they were not employed directly by the GP practice, they don’t really have any structured training as such they just develop with experience and develop their skills … Having some sort of a structured portfolio or pathway definitely helps with competence and safer practice” (PIP 11)*

Among those participants who had received formal PIP training, some reported that this did not provide them with the full range of skills needed in a GP practice setting, particularly when required to provide consultations to patients with multiple conditions.

*“There’s a lot of things that could be related to blood pressure which could be easily done had you as a pharmacist had better physical, clinical training … I think that that’s a little bit of barrier because we do as pharmacists lack those clinical skills” (PIP 6)*

Participants highlighted the importance of having access to clear national guidelines to carry out their roles safely and effectively as well as to demonstrate their value to the practice:

*“I believe you can make as long as you keep your interventions evidence based and up to date so it’s all about practicing evidence based medicine, which is why I always refer to the latest Guidelines and NICE [the National Institute for Health and Care Excellence]” (PIP 2)*

Self-learning, such as reading relevant documentation while considered to be beneficial, was often perceived to be time-consuming and challenging.

“If you haven’t got the correct training, you are just kind of sitting there trying to figure everything out for yourself … *It takes a lot longer to try and learn something by yourself.” (PIP 11)*

Participants also emphasised that adequate supervision and support by the practice general practitioners and other colleagues were especially important.

“Feedback and supervision are quite important as well so that you feel you are doing the right thing and meeting the practice’s expectation.” (PIP 10)

Some participants reported having formal induction or training systems at their practice which had helped them develop the necessary skills to perform required roles. These included, receiving mentorship from senior colleagues or having the opportunity to shadow a senior pharmacist in the practice, or having a culture of learning in which they are encouraged to develop as ACP (Advanced Clinical Practice) pharmacists.

“Having a mentor helped, having experience of sitting in with other colleagues did help as well, just to see what your consultation skills should be like and work on those.” (PIP 12)

“I feel that the integration has been far easier because the lead surgery is a training practice” (PIP 6)

##### Interpersonal skills of PIP

In addition to specialist knowledge, participants highlighted that good interpersonal skills, such as verbal communications or collaborative and relationship-building skills, was also essential to facilitate the PIPs’ integration into GP practice.

*“Taking the time to ask about friends, family, working relationships so that you actually become a nice colleague before you actually start moving on to do nice work. So you are already developing those relationships rather than trying to go in and implement change straight away. (PIP 1)*

“Softer skills are really important because they are also used to build a rapport with patients” (PIP 1)

Having informal conversations or giving more formal presentations to staff to raise awareness of their skills were described as facilitators of integration.

*“Not just talking to GPs but talking to receptionists, talking to Health Care Assistants, talking to Nurses, talking to Social Prescribers, talking to everybody so that they know what you can do what they can do and working with the team” (PIP 3)*

*“Very much about you being there and involving yourself in all the processes. So I don’t just work at my desk, I will try and mix with the nurses, I will speak to the doctors, I will go to the clinical meetings, I will present at clinical meetings and actively participate where I can.” (PIP 3)*

### Patients’ perception of their most recent consultation with the PIPs

Patients reported very high levels of satisfaction with all aspects of their appointments with PIPs except for the number of appointments they had with PIPs (Table 4).

**Table 4:** Patients' satisfaction with aspects of appointments with PIPs

Statements	VS or S (%)	Neutral (%)	NS or NAS (%)
The benefit of having a PIP appointment at the GP practice	72 (93.5%)	5 (6.5%)	0 (0%)
Support received from the PIP	73 (94.8%)	4 (5.2%)	0 (0%)
Spending enough time with the PIP during the appointment	74 (96.1%)	3 (3.9%)	0 (0%)
Ability of the PIP to help improving the condition	71 (92.2%)	6 (7.8%)	0 (0%)
Overall impression of visit	72 (93.5%)	5 (6.5%)	0 (0%)
PIP’s understanding of the patient’s point of view	74 (96.1%)	3 (3.9%)	0 (0%)
Number of appointments with PIPs at the GP practice	46 (59.8%)	25 (32.5%)	6 (7.8%)

*VS* very satisfied, *S* satisfied, *NS* not satisfied, *NAS* not at all satisfied, *GP *general practice, *PIPs* pharmacist independent prescribers

The majority of patients were confident with the PIP’s recommendations (97%, *n* = 75) and did not see the need to further consult a general practitioner after their appointment with the PIP (75%, *n* = 58). However, 14% of patients (*n* = 11) felt they still needed to consult a general practitioner after attending the PIP appointment.

A total of 62 open comments of patients related to the services provided by PIPs in GP practice were received. Table 5 shows the generated themes from open comments with supporting quotations.

**Table 5:** Identified benefits of services provided by PIPs with supporting quotations

Themes	Supporting quotations
Accessibility and convenience	“Less waiting time for an appointment” (Patient no. 47) “Ready accessibility to NHS services” (Patient no. 22)
Specialist expertise	“I find that pharmacist has more knowledge about medications than a GP” (Patient no. 50) “Peace of mind on the medication being taken over long period of time. Time to discuss any concerns regarding medication” (Patient no. 46)
Longer consultation/appointments	“Unrushed, comprehensive discussion of condition” (Patient no. 3) “Have more time with a health care professional” (Patient no. 48) “They have more time to go through the different types of medications and side effects” (Patient no. 50)
Attention and/or helpfulness	“She was very helpful and provided us with all the important information we needed” (Patient no. 44) “Very concerned about your person” (Patient no. 27)
Relieving pressure	“Not having to a see a GP for non-urgent appointments” (Patient no. 23) “Saves GP time” (Patient no. 48)

## Discussion

This study explored the perspectives of PIPs regarding the integration of PIPs into practice when providing services in GP settings and patient satisfaction with the services provided. Most PIPs reported gradually becoming better integrated into their practices and feeling supported by their colleagues. Key factors impacting the successful implementation of the PIP workforce into GP practice were mainly centred on lack of understanding of pharmacists’ roles within multidisciplinary teams and to some extent, resistance to change from the clinical practice team. Given that there is no standardized training pathways for pharmacists aspiring to work in GP practice, the lack of clinical competence and interpersonal skills required to work in GP practice could also affect PIPs’ integration in GP practice. Patients’ views of having a PIP appointment at their GP practice were positive overall. Almost all the patients were confident about the PIPs’ recommendations despite some patients reporting that they still needed to further consult a general practitioner after their appointments.

Some of the study findings are consistent with the current evidence regarding the barriers that PIPs face when integrating into GP practice. Our findings suggest that a lack of a clearly defined role for PIPs in GP practice often leads to a narrow scope for practice, a limited number of opportunities to take on more patient-facing roles coupled with resistance from other clinical staff regarding the current role. Similar findings have also been identified from previous studies on GP pharmacists' practise in England [17, 20, 25], Australia [25, 38, 39], New Zealand [40], and Canada [41, 42]. Moreover, a qualitative study that reported on a localised training programme identified similar issues related to the clarity of the pharmacist's role in GP practices in England [21]. Previous studies suggest that having a more experienced clinical pharmacist in GP practice to mentor and supervise newly employed pharmacists could overcome issues with role clarity and facilitate the establishment of a professional identity [26, 27]. Duncan et al. [43] study, which investigated the barriers and facilitators in collaborative working between general practitioners and GP pharmacists in the UK, found that practitioners who had previously worked with a GP pharmacist reported a higher appreciation of their professional expertise and knowledge than practitioners who had not previously worked with a pharmacist. Furthermore, GP pharmacists felt their role was more efficiently utilised when they had a good relationship with a clinical practitioner [43].

Our study confirms findings from previous studies which show that PIPs face challenges with issues/queries on areas outside of their clinical competence [17, 20, 44, 45]. Pharmacist independent prescribers in the UK tend to be trained in a specific area, while their role in GP practice is quite broad and general, which limits their knowledge of prescribing outside their clinical area of competence. Our findings also demonstrate that PIPs can expand their clinical roles with time provided they are given the opportunity to take on more roles or responsibilities under the mentorship and supervision of other senior staff [27, 46, 47]. There is a need for structured experiential learning training programmes in GP practice that would expose PIPs to a wider spectrum of clinical case scenarios [21].

Patients in this study were satisfied with the care provided by PIPs. This is in parallel to the findings of previous studies that suggested that patients with chronic diseases reported high levels of acceptance of care provided by non-medical independent prescribers [48–50].

In contrast to the findings from previous research [51, 52], findings in this study indicate that some patients desired more appointments with PIPs at GP practices. Patients acknowledged having more time to discuss their conditions, medications or concerns in their appointments with their PIP. Previous evidence has also highlighted that patients reported to have longer consultation times with non-medical prescribers, and this was generally viewed positively by the patients [49, 53–55]. Another study by Gerard et al. [56] found that the consultation length had no impact on patient satisfaction with PIPs in a GP setting, while attributes relating to patient–professional interaction had an impact on the management of their medical condition.

To the authors’ knowledge, this is the first mixed-methods study in England that has explored the implementation of PIPs’ integration into GP practice and patients’ perspectives about PIP consultations. The sample size for interviews is similar to those in previous qualitative studies [57] and the interviews conducted were sufficient to achieve data saturation [58]. The questionnaire sample size was relatively small, since in-person consultations in GP practices were halted due to the COVID-19 pandemic. pharmacist independent prescribers distributed the questionnaire to their patients, which could have introduced an element of recruitment or response bias. However, the participant information sheet offered reassurance of anonymity and confidentiality, with the emphasis that the intention of the study was to assess the services provided rather than evaluate the practice of individual PIPs.

Given the use of convenience sampling in the study, the authors acknowledge the potential for self-reporting bias, social desirability bias and recall bias as well as the researchers’ bias in the collection, analysis and interpretation of the data. Nevertheless, the sample consisted of PIPs with varied work experience and from different geographical GP settings across England. Patients were from different GP practices based on different geographical areas, potentially augmenting the generalisability of the study findings. Moreover, reflexivity was considered to minimise the researcher's bias during the interviews and analysis.

## Conclusion

The findings of this study suggest that understanding PIPs’ roles as well as facilitators and barriers to working in GP practice is a prerequisite for successful integration. More research is needed around optimising the training, education and integration of PIPs in GP practice to ensure they are competent and confident to take on new roles.

## Supplementary Information

**Additional file 1.** The developed survey for patients’ satisfaction with pharmacist independent prescriber consultations in general practice.

## Data Availability

The data sets used and/or analysed during this study are available from the corresponding author on reasonable request.
