# Supplementary material for: Integration of pharmacist independent prescribers into general practice: a mixed-methods study of pharmacists’ and patients’ views
Source: J Pharm Policy Pract. 2023 Jan 19;16:10. doi: 10.1186/s40545-023-00520-9 (PMC9851587; doi:10.1186/s40545-023-00520-9)
Supplement: Supplementary file 1 — Additional file 1. The developed survey for patients’ satisfaction with pharmacist independent prescriber consultations in general practice. [file 40545_2023_520_MOESM1_ESM.docx]

# General Information

1. **Gender:**

Male Female Prefer not to say

1. **Age ________________ years**
2. **What is the main condition that made you attend the pharmacist clinic today?**

High blood glucose

High blood presure

Dyslipidaemia (High cholesterol/ High triglyceride)

Other, *please state* ______________________

1. **How long was your current appointment with the pharmacist at the GP surgery?**

**____________________ Minutes**

1. **Pharmacist advice and intervention given to you was about:** *Please tick* ***all that apply***

Disease

Medication

Lifestyle

Other, *please state* ______________________

1. **How many appointments have you had previously with pharmacists at the GP surgery since 2015?**

**____________________ Appointments**

# YOUR SATISFACTION

**Please evaluate the services that provided by the pharmacist at GP surgery.** *Please mark your answer by placing a tick in the box.*

| **Please answer each of the following questions on a scale of 1 to 5.** | **Not at all satisfied 1** | **Fair 2** | **Ok**  **3** | **Good 4** | **Very much satisfied 5** |
| --- | --- | --- | --- | --- | --- |
| 1. ****Understanding of the pharmacist to your point of view**** |  |  |  |  |  |
| 1. ****Support you received from the pharmacist**** |  |  |  |  |  |
| 1. ****The ability of the pharmacist to help in improving your condition**** |  |  |  |  |  |
|  | **Not at all satisfied 1** | **Fair 2** | **Ok**  **3** | **Good 4** | **Very much satisfied 5** |
| 1. ****Overall impression at your visit**** |  |  |  |  |  |
| 1. ****Spending enough time with the pharmacist during your appointment**** |  |  |  |  |  |
| 1. ****The benefit of having a pharmacist clinic at GP surgery**** |  |  |  |  |  |
| 1. ****Number of appointments you had with Pharmacists at GP surgery**** |  |  |  |  |  |

1. **Are you confident of the recommendations provided to you by the pharmacist?**

Yes No Not sure

1. **Do you think that attending the pharmacist clinic would improve your condition?**

Yes No Not sure

1. **After attending the pharmacist clinic,** **do you still need to consult a GP doctor?**

Yes No Not sure

# YOUR VIEWS

1. **What is the best part about the services provided by pharmacists at GP surgeries?**
2. **Do you have other comments about the service provided by the practice pharmacist? If yes, please write your comments below.**

**THANK YOU FOR COMPLETING THIS QUESTIONNAIRE**
